# Polycistronic trypanosome mRNAs are a target for the exosome

**DOI:** 10.1016/j.molbiopara.2016.02.009

**Published:** 2016

**Authors:** Susanne Kramer, Sophie Piper, Antonio Estevez, Mark Carrington

**Affiliations:** a Biozentrum, Julius-Maximilians Universität Würzburg, Am Hubland, 97074 Würzburg, Germany; b Department of Biochemistry, University of Cambridge, Tennis Court Road, Cambridge CB2 1QW, UK; c Instituto de Parasitología y Biomedicina “López-Neyra”, IPBL N-CSIC, Parque Tecnológico de Ciencias de la Salud, Avda. del Conocimiento, s/n.18100 Armilla, Granada, Spain

**Keywords:** *Trypanosoma brucei*, Exosome, NMD, Polycistronic mRNA, *trans*-splicing, Trypanosomes

## Abstract

Eukaryotic cells have several mRNA quality control checkpoints to avoid the production of aberrant proteins. Intron-containing mRNAs are actively degraded by the nuclear exosome, prevented from nuclear exit and, if these systems fail, degraded by the cytoplasmic NMD machinery. Trypanosomes have only two introns. However, they process mRNAs from long polycistronic precursors by *trans*-splicing and polycistronic mRNA molecules frequently arise from any missed splice site. Here, we show that RNAi depletion of the trypanosome exosome, but not of the cytoplasmic 5′-3′ exoribonuclease XRNA or the NMD helicase UPF1, causes accumulation of oligocistronic mRNAs. We have also revisited the localization of the trypanosome exosome by expressing eYFP-fusion proteins of the exosome subunits RRP44 and RRP6. Both proteins are significantly enriched in the nucleus. Together with published data, our data suggest a major nuclear function of the trypanosome exosome in rRNA, snoRNA and mRNA quality control.

## Highlights

• RNAi depletion of exosomal subunits causes accumulation of oligocistronic mRNAs.
• RNAi depletion of the 5′-3′ exoribonuclease XRNA or UPF1 has no effect on oligocistronic mRNAs.
• eYFP fusions of the exosome subunits RRP44 and RRP6 localize to the nucleus.

## Graphical abstract

**Figure:**
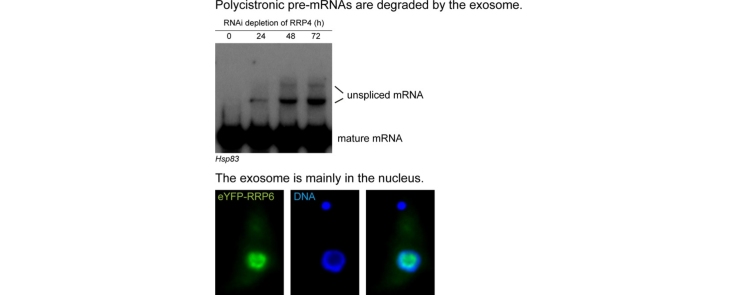

## 1 Introduction, results and discussion

Splicing of pre-mRNAs is not 100% efficient. The translation of intron-containing mRNAs would be harmful to the cell and eukaryotic cells have developed several control systems that act in parallel to avoid the production of aberrant proteins. One major system is the active retention of unspliced mRNAs in the nucleus with several components of the nuclear pores being involved [1], [2]. If this system fails, intron-containing mRNAs are recognized and degraded by the cytoplasmic nonsense mediated decay (NMD) system. In yeast for example, many mRNA precursors accumulate in strains carrying mutations of the two essential NMD proteins Upf1p or Xrn1p [3]. A third system is the active degradation of intron-containing mRNAs by the nuclear exosome/TRAMP (Trf–Air–Mtr4 polyadenylation) complex, with the processive 3′-5′ *exo*- and *endo*ribonuclease Dis3p/Rrp44 being the responsible catalytic component [1]. In yeast, both the spliceosome and the exosome compete for intron-containing mRNAs [4]. This results in the degradation of more than half of all intron-containing mRNAs before they can enter the spliceosomal machinery [4]: a high energetic price to ensure mRNA quality. There is good evidence for the existence of a similar system in trypanosomes from a recent transcriptome-wide analysis of trypanosome mRNA decay pathways [5].

Only two genes in *Trypanosoma brucei* contain *cis*-introns. However, the parasites encounter another problem of mRNA quality control instead: the accumulation of di- and oligocistronic mRNAs precursors due to inefficient *trans*-splicing. Trypanosomes have an unusual way of transcription: tens to hundreds of genes are co-transcribed and subsequently processed to mature mRNAs by the addition of the capped, 39 nucleotide long mini-exon from the spliced leader mRNA to the 5′ end. This *trans*-splicing is coupled to the polyadenylation of the mRNA from the upstream gene [6]. Like *cis*-splicing, *trans*-splicing is not 100% efficient. Some splice sites are missed, resulting in the formation of di- or oligocistronic mRNA molecules that are present in the nucleus and partially even in the cytoplasm [7], [8], [9]. An accumulation of oligocistronic mRNAs is potentially harmful: mRNAs encoded by neighbouring genes are not usually related to each other and are likely to contain mixed regulatory elements. The consequence would be changes in the post-transcriptional regulation of gene expression. There is some evidence for the presence of an active mechanism to keep unspliced mRNAs in the nucleus, as partially processed tubulin mRNAs are more concentrated in the nucleus than in the cytoplasm [10]. Moreover, the half-life of tubulin dicistrons is significantly shorter than the half-life of mature tubulin mRNA, indicating that an active mechanism for the removal of unspliced mRNAs may exist in trypanosomes [7]. Trypanosomes have an exosome that is essential [11] as well as orthologues to all three components of the TRAMP complex [12], [13]. The best characterized nuclear function of the trypanosome exosome/TRAMP complex is the trimming of the 5.8S rRNA precursors [11], [13]. In addition, there is evidence for an involvement in snoRNA processing [14]. Both are expected functions of eukaryotic exosomes [15]. Whether the trypanosome exosome also has cytoplasmic functions in mRNA quality control is uncertain [16]. There are changes in mRNA levels upon RNAi depletion of exosome components, but these could also be due to secondary effects caused by the growth arrest [14], [17]. Trypanosomes also have an orthologue to UPF1, the ATP dependent RNA helicase required for NMD, but it still remains unclear, whether they possess a canonical NMD pathway: the introduction of a premature termination codon causes the expected destabilization of both an endogenous and a reporter mRNA, but this destabilization is not dependent on UPF1 [18]. In contrast, the cytoplasmic 5′-3′ exoribonuclease XRNA, the trypanosome orthologue to yeast XRN1, is essential and its depletion causes global stabilization of mRNAs with a preference for short lived mRNAs [19]. Here, we have examined any potential contribution of the trypanosome exosome, of the trypanosome NMD pathway and of XRNA to the removal of unspliced mRNA precursors and thus to mRNA quality control.

**Fig. 1:**
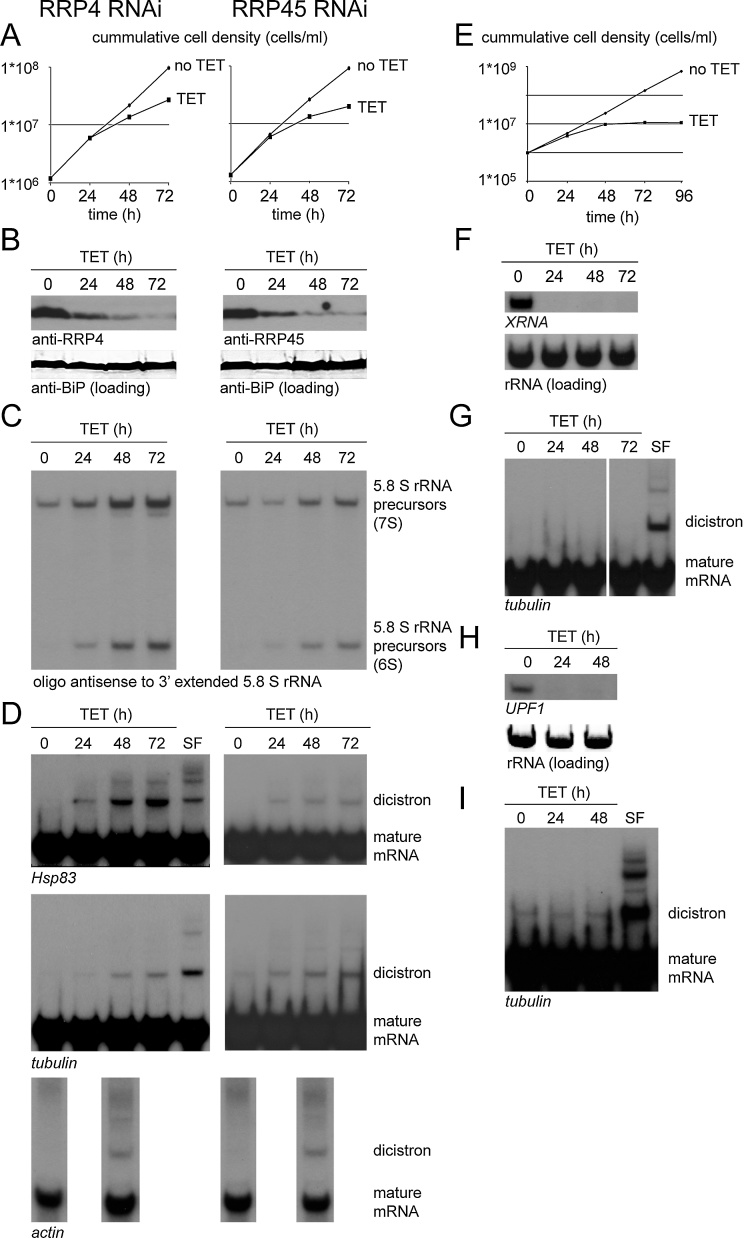
Inducible RNAi depletion of RRP4, RRP45, XRNA and UPF1. RNAi was induced by tetracycline (TET). All experiments shown in this figure, with the exception of the actin northern blot in (D) were also done with a second RNAi clone, with similar results (data not shown). Northern and western blots were done according to standard procedures. All northern blots were loaded with equal amounts of total RNA. (A) Growth in the absence and presence of RNAi depletion of RRP4 (left) or RRP45 (right). (B) Western blots: RRP4 and RRP45 proteins were detected on a western blot at different time-points after RNAi induction using previously described polyclonal antiserum [11]. BiP served as loading control. (C) Northern blots: detection of 5.8S rRNA maturation precursors by an oligo antisense to the 3′ extended 5.8 S rRNA (5′-GTTTTTATATTCGACACTG-3′) at different time-points after RRP4 or RRP45 RNAi induction. For loading, compare mature mRNAs on the northern blots in D, which contain the same mRNA samples. (D) Northern blots: detection of *Hsp83*, α–*tubulin* and *actin* at different time-points after RRP4 and RRP45 RNAi induction. Mature and dicistronic mRNAs are indicated. As a control, RNA of cells treated with sinefungin (SF) for one hour is loaded. *Hsp83*, *tubulin* and *actin* probes were antisense to the complete ORF sequence of the respective genes. (E) Growth in the absence and presence of RNAi depletion of XRNA. (F) Northern blot: detection of *XRNA* mRNA in RNA samples taken over a time-course of XRNA depletion. rRNA served as loading control. Nucleotides 665-1794 of the *XRNA* open reading frame were used as a probe. (G) Northern blot: detection of α*–tubulin* at different time-point after RNAi depletion of XRNA. As a control, RNA of cells treated with sinefungin (SF) for one hour is loaded. (H) Northern blot: detection of *UPF1* mRNA in RNA samples taken over a time-course of UPF1 depletion. rRNA served as loading control. The C-terminal 823 nucleotides of the UPF1 ORF were used for probing. (I) Northern blot: detection of α–*tubulin* at different time-point after RNAi depletion of XRNA. As a control, RNA of cells treated with sinefungin (SF) for one hour is loaded. The blot was over-exposed on purpose to stress the absence of precursor mRNAs.

First, the involvement of the exosome in mRNA precursor degradation was tested. Two components of the trypanosome exosome, the S1 subunit RRP4 and the RNAse PH subunit RRP45, were individually depleted by RNAi knockdown using previously described RNAi plasmids [11] in Lister 427 procyclic trypanosomes containing a TetR transgene after integration of pSPR2 [20]. With both RNAi experiments, a reduction in growth (Fig. 1A), a reduction in RRP4 and RRP45 proteins (Fig. 1B) and an accumulation of 5.8S rRNA precursors (Fig. 1C) was observed. These results are in agreement with previously published data [11] and validate the knock-downs. Three different mRNAs were chosen for the analysis of precursor accumulation after induction of RNAi: *HSP83*, α–tubulin and actin. All three mRNAs are encoded by multigene families arranged in tandem arrays [21]. The tandem arrangement facilitates the detection of di- and oligocistronic mRNAs as these can accumulate from the exclusion of more than one splice site. For both α–tubulin and *HSP83,* RNA molecules larger than the mature mRNAs became detectable or increased within 24 h of depletion of either RRP4 or RRP45 (Fig. 1D). There was a further increase between 48 and 72 h (Fig. 1D). The size of these large RNAs was the same as RNAs that result from the inhibition of *trans*-splicing with sinefungin (SF) [22], indicating they resulted from incomplete *trans*-splicing (Fig. 1D). For actin, RNA samples were analysed after 48 h of RNAi induction only: incompletely spliced mRNAs were present after both RRP4 and RRP45 depletion (Fig. 1D). The accumulation of incompletely spliced mRNAs in response to the depletion of exosome components strongly suggests a participation of the exosome in the degradation of incompletely spliced mRNAs.

Next, the involvement of cytoplasmic components in the degradation of mRNA precursors was tested. Induction of XRNA RNAi resulted in a decrease in XRNA mRNA (Fig. 1F) and a cessation of growth (Fig. 1E) in agreement with previously published data [23]. Incompletely spliced tubulin RNAs were not detected over a time course after induction of XRNA RNAi (Fig. 1G) indicating that the cytoplasmic 5′-3′ degradation pathway plays either no or only a minor role in the degradation of precursor mRNAs. Induction of UPF1 RNAi resulted in decrease in UPF1 mRNA (Fig. 1H), and this had no effect on cell proliferation (data not shown). No increase in incompletely spliced RNAs was detectable. These data provide evidence that neither XRNA nor UPF1 are involved in the degradation of incompletely spliced mRNAs.

**Fig. 2:**
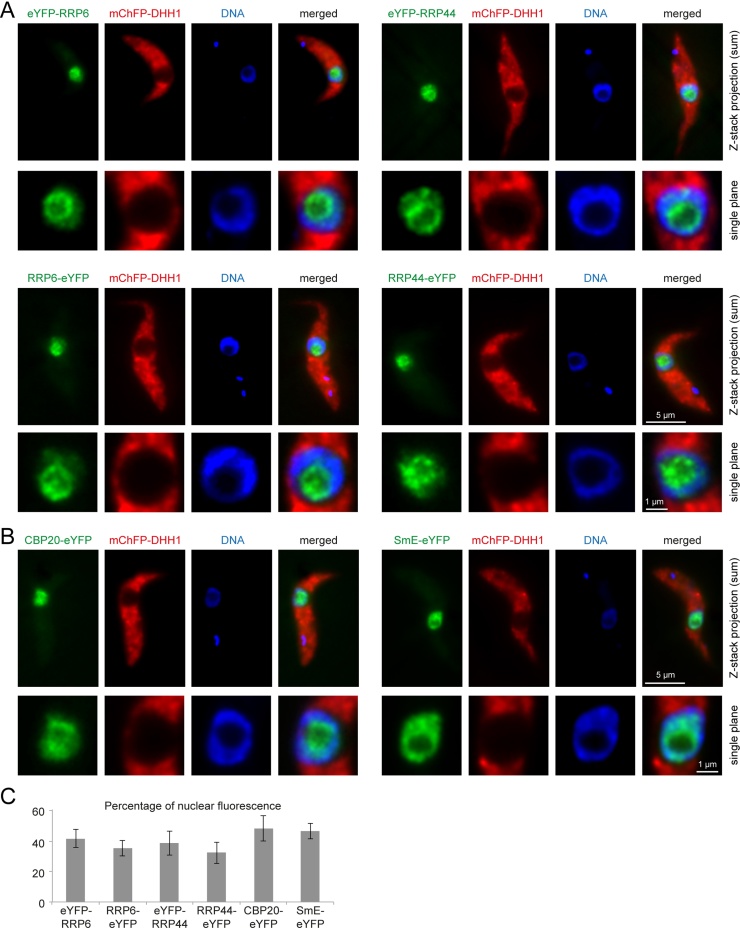
Localization of N and C-terminal eYFP fusions of RRP6 and RRP44. Two nuclear control proteins (CBP20 and SmE) served as controls. (A + B) Z-stacks (100 images, 100-nm spacing) were recorded with a custom-built TILL Photonics iMIC microscope equipped with a 100×, 1.4 numerical aperture objective (Olympus, Tokyo, Japan) and a sensicam qe CCD camera (PCO, Kehlheim, Germany); deconvolved using Huygens Essential software (SVI, Hilversum, The Netherlands). For each cell line, one representative fluorescent cell is shown as a Z-stack projection (method sum slices). In addition, the nucleus of a deconvolved single plane image is shown enlarged. (C) For each eYFP fusion protein, the percentage of fluorescence in the nucleus was quantified from the Z-stack projections of deconvolved images of at least 16 cells. Error bars indicate standard deviations.

The data above indicate that the trypanosome exosome is necessary for the degradation of incompletely spliced mRNAs. This adds one further function to the nuclear exosome in trypanosomes. The localization of the exosome still remains unclear in trypanosomes. Initial non-quantitative fractionation studies showed a localization of RRP4, RRP44 and RRP45 to both the cytoplasm and the nucleus [11]. A later fractionation study found the majority of RRP4, RRP44, RRP45 and RRP6 localized in the cytoplasm [24]. The same study also used anti-protA to localize TAP-tagged RRP4 and this appeared to be more concentrated in the nucleus compared to the cytoplasm, particularly at the edge of the nucleolus. Antiserum raised to RRP6 gave speckled signal throughout the cell [24]. Fractionation methods have the problem that proteins can leak out of the nucleus and immunofluorescences can be misleading. The localization of RRP44 and RRP6 was investigated using eYFP-tagged transgenes expressed from the endogenous loci [25]. Both C- and N-terminally tagged fusion proteins of RRP44 and RRP6 were used to minimize the risk of potential mislocalisation caused by the eYFP tag. The cell lines also expressed an N-terminal mCherry fusion protein of the Dead box RNA helicase DHH1, a marker for cytoplasmic RNA granules that is mainly localized to the cytoplasm. eYFP fusions of the nuclear cap binding protein CBP20 and the spliceosomal protein SmE served as controls for nuclear proteins. In all cells, both N- and C-terminal eYFP fusions of RRP44 and RRP6 were highly concentrated in the nucleus, with a slight enrichment at the edge of the nucleolus, which is here detected by the absence of DAPI staining (Fig. 2A). This localization was similar to the previously published localization of RRP4 [24]. As expected, CBP20 and SmE also localized to the nucleus (Fig. 1B). SmE was mainly excluded from the nucleolus; the expression level of CBP20-eYFP was too low to be certain about its subnuclear localization (Fig. 1B). The percentage of nuclear fluorescence was quantified from Z-stack projections of at least 16 cells for each of the cell lines. There were only minor differences between the cells expressing eYFP fusions of the nuclear control proteins and the cells expressing eYFP fusions of the exosome proteins: the control cells had 46% (SmE) and 48% (CBP20) nuclear fluorescence, the cell lines expressing exosome proteins had between 32% and 42% (Fig. 1C). The quantification of fluorescence underestimates the true fraction of the proteins in the nucleus, because trypanosomes have some auto-fluorescence.

These data are evidence for the trypanosome exosome being mostly in the nucleus, with enrichment at the edge of the nucleolus. The localization of a minor fraction to the cytoplasm cannot be excluded. We found no evidence for a localization of either RRP44 or RRP6 to trypanosome RNA granules (Fig. S1). These included starvation stress granules induced by incubation in PBS and nuclear periphery granules induced by the inhibition of *trans*-splicing (Fig. S1). There was also no localization of exosome subunits to heat shock stress granules; however, heat shock also caused a major relocalisation of RRP44 and RRP6 to the cytoplasm (Fig. S2). Since there was a similar relocalisation of SmE and CBP20 to the cytoplasm, the physiological relevance remains unclear and this observation was not further examined.

The problem of accumulation of oligocistronic mRNAs is unique to the relatively small group of eukaryotes that perform *trans*-splicing; only kinetoplastids completely rely on it. Here we show that the trypanosome exosome rather than the cytoplasmic NMD pathway actively degrades such wrongly processed RNA precursor molecules. RNAi depletion of exosomal subunits, but not of XRNA or UPF1 causes precursor accumulation. This is unlikely a secondary effect of the growth arrest, because XRNA depletion causes an even more severe growth arrest without any precursor accumulation. In addition to the previously described functions of the trypanosome exosome in rRNA and snoRNA quality control, we here add one further function in mRNA quality control. How are oligocistronic mRNAs recognized by the exosome? This question has not been answered for intron-containing mRNAs of any eukaryotes [15]. Two scenarios are possible: RNA targets could be specifically recognized by the exosome via a yet unknown, specific exosome specificity factor (ESF) that marks the molecule as unspliced. Perhaps decay is then initiated by the endonuclease activity of the PIN domain of RRP44. Alternatively, the exosome/TRAMP complex could target RNA molecules non-specifically. Unspliced RNA molecules would be preferentially degraded because they are prevented from leaving the nucleus and thus are longer exposed to the degradation machinery. In this model, the mRNA quality control would entirely reside in the nuclear export control system with the exosome being the executioner. One open question in the trypanosome field has been the localization of the exosome. Our data strongly suggest that the majority of the exosome is in the nucleus, rather than in the cytoplasm, as we do not find major differences in nuclear localization between exosomal proteins and proteins with expected nuclear localizations. We cannot, of course, exclude a minor localization to the cytoplasm. All three established functions of the trypanosome exosome in rRNA and snoRNA control and in mRNA precursor degradation fit to a mainly nuclear localization.
