# Supplementary material for: Polycistronic trypanosome mRNAs are a target for the exosome
Source: Mol Biochem Parasitol. 2016 Jan-Feb;205(1-2):1–5. doi: 10.1016/j.molbiopara.2016.02.009 (PMC4850246; doi:10.1016/j.molbiopara.2016.02.009)
Supplement: Supplementary file 1 [file mmc1.pdf]

Figure S1

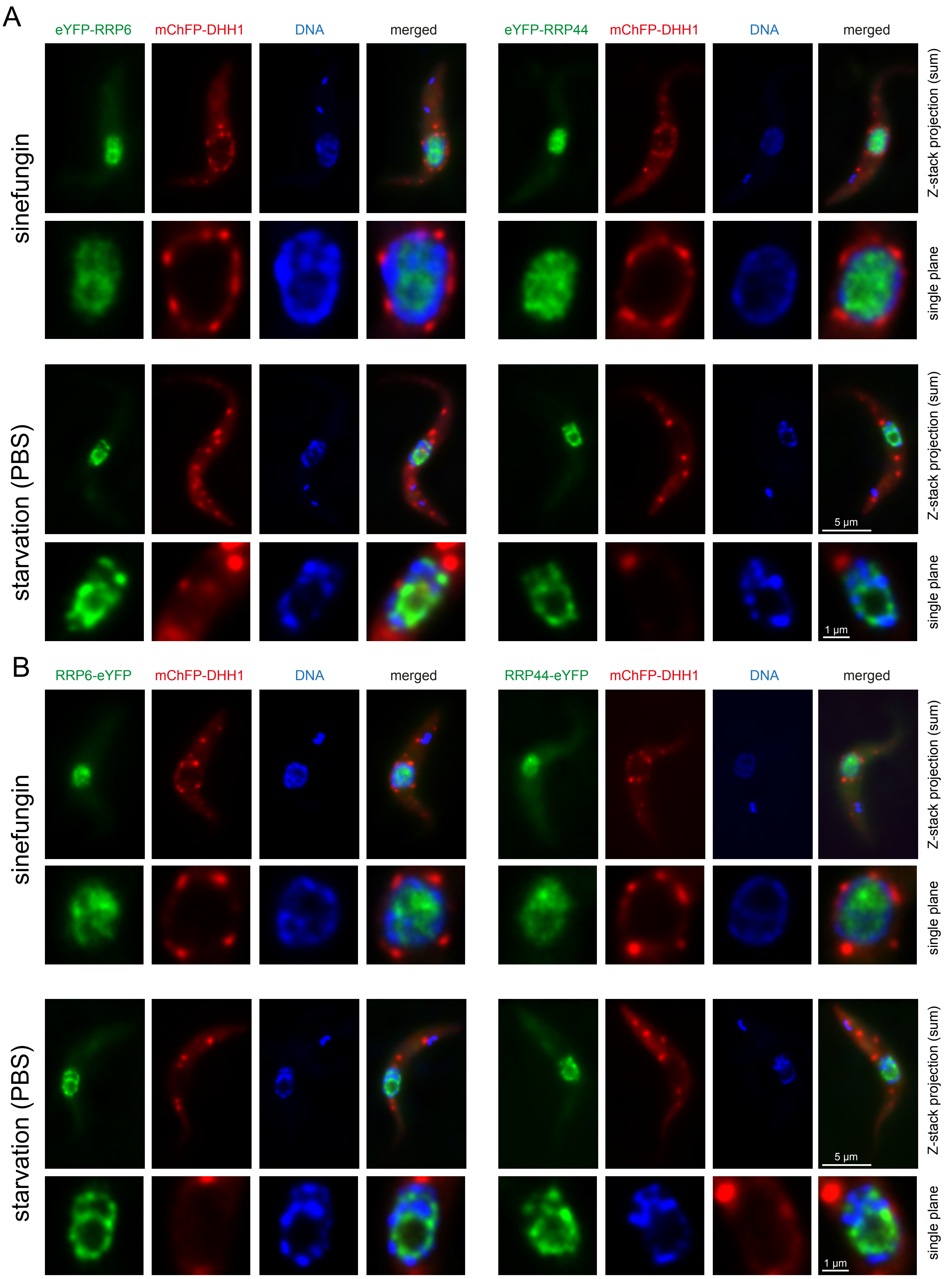
A
eYFP-RRP6
mChFP-DHH1
DNA
merged
eYFP-RRP44
mChFP-DHH1
DNA
merged
sinefungin
starvation (PBS)
Z-stack projection (sum)
single plane
Z-stack projection (sum)
single plane
5 µm
1 µm
B
RRP6-eYFP
mChFP-DHH1
DNA
merged
RRP44-eYFP
mChFP-DHH1
DNA
merged
sinefungin
starvation (PBS)
Z-stack projection (sum)
single plane
Z-stack projection (sum)
single plane
5 µm
1 µm

Figure S2

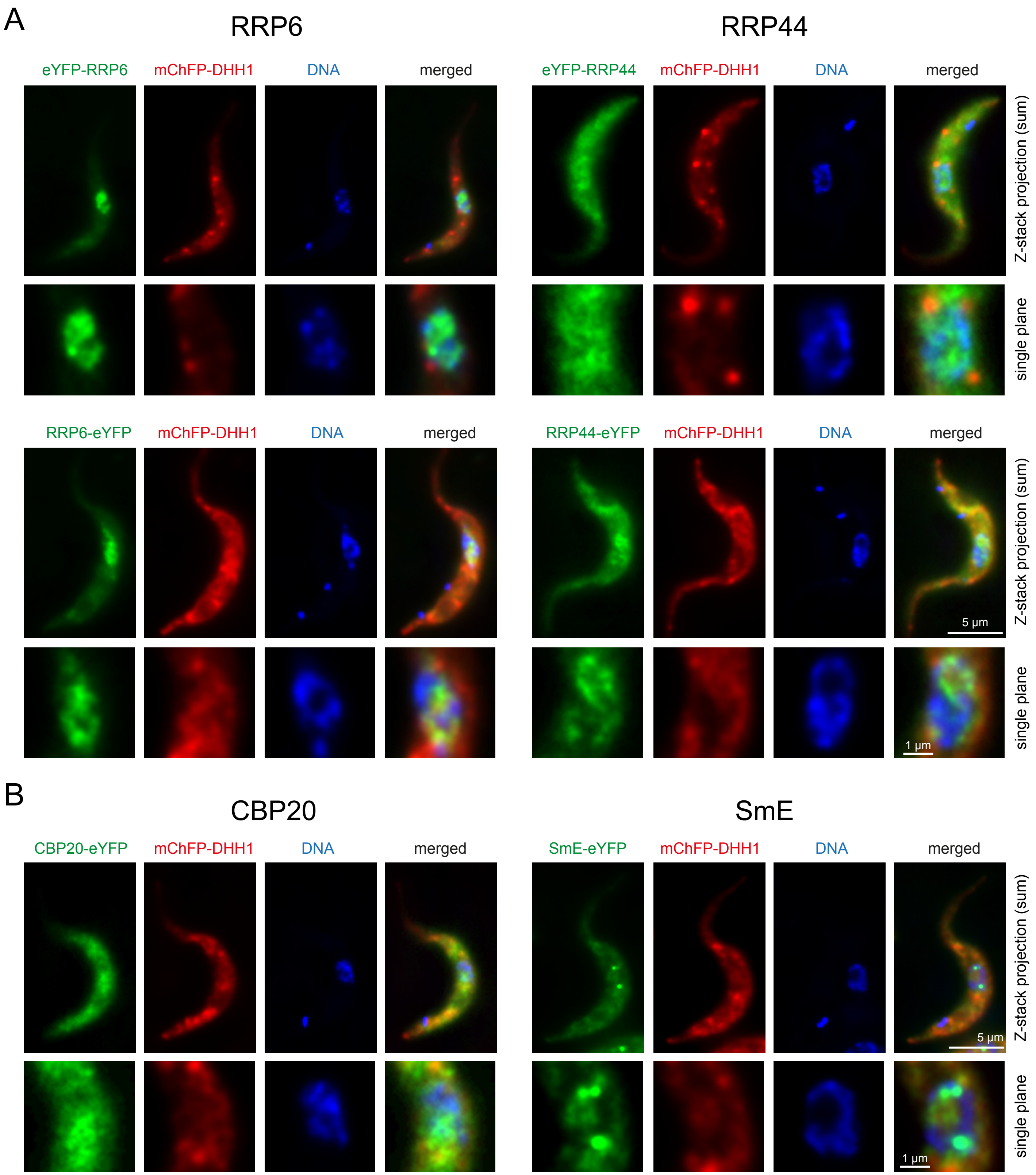
A
RRP6
RRP44
eYFP-RRP6
mChFP-DHH1
DNA
merged
eYFP-RRP44
mChFP-DHH1
DNA
merged
Z-stack projection (sum)
single plane
RRP6-eYFP
mChFP-DHH1
DNA
merged
RRP44-eYFP
mChFP-DHH1
DNA
merged
5 µm
1 µm
Z-stack projection (sum)
single plane
B
CBP20
SmE
CBP20-eYFP
mChFP-DHH1
DNA
merged
SmE-eYFP
mChFP-DHH1
DNA
merged
5 µm
1 µm
Z-stack projection (sum)
single plane

Figure legends

Fig. S1: Localization of N and C-terminal eYFP fusions of RRP6 and RRP44 at starvation or sinefungin treatment

Cells expressing mChFP-DHH1 and RRP6 or RRP44 as either N-terminally **(A)** or C-terminally **(B)** tagged eYFP fusion protein were treated with sinefungin (2 µg/ml, 1 hour) or starved (cultured in PBS for 2 hours).

Z-stacks (100 images, 100-nm spacing) were recorded with a custom-built TILL Photonics iMIC microscope equipped with a 100×, 1.4 numerical aperture objective (Olympus, Tokyo, Japan) and a sensicam qe CCD camera (PCO, Kehlheim, Germany) and deconvolved using Huygens Essential software (SVI, Hilversum, The Netherlands). For each cell line, one representative fluorescent cell is shown as a Z-stack projection (method sum slices). In addition, the nucleus of a deconvolved single plane image is shown enlarged.

Fig. S2: Localization of N and C-terminal eYFP fusions of RRP6 and RRP44 at heat shock treatment

Cells expressing mChFP-DHH1 and RRP6 or RRP44 as either N-terminally or C-terminally tagged eYFP fusion protein **(A)** and, as a control, cells expressing CBP20-eYFP or SmE-eYFP **(B)** were treated with heat shock (2 hours, 41°C).

Z-stacks (100 images, 100-nm spacing) were recorded with a custom-built TILL Photonics iMIC microscope equipped with a 100×, 1.4 numerical aperture objective (Olympus, Tokyo, Japan) and a sensicam qe CCD camera (PCO, Kehlheim, Germany) and deconvolved using Huygens Essential software (SVI, Hilversum, The Netherlands). For each cell line, one representative fluorescent cell is shown as a Z-stack projection (method sum slices). In addition, the nucleus of a deconvolved single plane image is shown enlarged.
